# The impact of contamination bias when replicating maltreatment effects on child behavior problems

**DOI:** 10.1371/journal.pone.0358837

**Published:** 2026-10-06

**Authors:** Anneke E. Olson, John M. Felt, Noah Greifer, Zachary F. Fisher, Kenneth A. Shores, Nilam Ram, Chad E. Shenk

**Affiliations:** 1 National Crime Victims Research and Treatment Center, Medical University of South Carolina, Charleston, South Carolina, United States of America; 2 Center for Healthy Aging, Pennsylvania State University, University Park, Pennsylvania, United States of America; 3 Institute for Quantitative Social Science, Harvard UniversityCambridge, Massachusetts, United States of America; 4 Department of Psychology and Neuroscience, University of North Carolina, Chapel Hill, North Carolina, United States of America; 5 College of Education and Human Development, University of Delaware, Newark, Delaware, United States of America; 6 Department of Communications, Stanford University, Stanford, California, United States of America; 7 Department of Psychology, Stanford University, Stanford, California, United States of America; 8 Department of Psychology, University of Rochester, Rochester, New York, United States of America; 9 Mt. Hope Family Center, University of Rochester, Rochester, New York, United States of America; Universitat Autonoma de Barcelona, SPAIN

## Abstract

**Background:**

Contamination, when members of a comparison condition receive or are exposed to a treatment under investigation, can bias the direction, statistical significance, and magnitude of treatment effects.

**Objective:**

This pre-registered study examined whether contamination biased estimates when replicating child maltreatment effects on two established outcomes, externalizing and internalizing behavior problems.

**Methods:**

A multi-wave, U.S. national probability sample of children and families involved with child protective services (*N* = 4000; Age = 0–17.5; Sex = 48% Female; Race = 60% White; Ethnicity = 30% Hispanic). Child maltreatment and comparison conditions were established using official case records and contamination was identified as caregiver-reported maltreatment for those children assigned to the comparison condition. Outcomes were 6-month frequencies of externalizing and internalizing behaviors, represented in *T*-scores, three years post-maltreatment exposure.

**Results:**

The prevalence of contamination in this study was 96%. Propensity score analyses that ignored contamination revealed that maltreatment exposure had no impact on externalizing behaviors (*b* = 0.28, *p* = .746) or internalizing behaviors (*b* = 0.27, *p* = .744), failing to replicate prior research. However, propensity score analyses that corrected contamination replicated prior research by generating estimates that were in the expected directions, statistically significant, and moderate to large in effect magnitude for externalizing (*b* = 8.72, *p* = .002, *SMD* = 0.74) and internalizing behavior problems (*b* = 9.96, *p* < .001, *SMD* = 0.90).

**Conclusion:**

Correcting contamination has the potential to improve the replication of child maltreatment effects, strengthening the generalizability of results.

## Introduction

Contamination is a methodological phenomenon that occurs in both randomized and observational research when individuals assigned to a control condition receive or are exposed to a treatment under investigation [1,2]. The presence of contamination poses a threat to the estimation of treatment effects, which include environmental exposures, because observed levels of the outcome for those in a control condition will more closely approximate levels of the same outcome for those in a treatment condition [3]. This results in a bias that can affect the direction of reported treatment effects, particularly under high levels of contamination, as well as weaken the statistical significance and magnitude of between-group differences [4–8]. Contamination therefore raises the possibility of scientific discovery and replication failures because some unknown proportion of individuals in a control condition received or were exposed to the treatment. Highlighting the presence and impact of contamination bias on treatment effects, and devising methods for correcting such bias, therefore has the potential to promote scientific discovery and reduce the frequency of replication failures – key features for promoting the reproducibility and generalizability of randomized and observational research [9–11].

Unfortunately, a lack of awareness about the presence and impact of contamination has resulted in a lack of solutions for correcting resulting bias, particularly in observational designs when random assignment to treatment and control conditions is unethical. This challenge is particularly relevant when estimating the effects of child maltreatment, an act of commission or omission by a caregiver resulting in harm or risk for harm toward an individual under the age of eighteen [12]. Child maltreatment is a highly-prevalent, worldwide public health concern [13–15] affecting a range of pediatric [16–18] and adulthood health outcomes [19–21]. Contamination occurs in child maltreatment research when members of a comparison condition have been exposed to child maltreatment, either prior to study enrollment or during longitudinal follow-up [22]. In this case, the “treatment” that is under investigation is an environmental exposure, “child maltreatment”, and the term, “control condition”, is replaced by the equivalent yet more commonly used term, “comparison condition”. Prior research has demonstrated a wide range in the prevalence of contamination in child maltreatment research, including samples from the general population as well as the child welfare population, where as many as 18%−67% of individuals in a comparison condition have experienced child maltreatment [23–26]. When ignored, contamination can bias the estimation of maltreatment effects by failing to detect statistically significant differences and truncating effect size magnitudes for a variety of outcomes, such as psychiatric disorders, teen birthrates, obesity, and cigarette use [27,28]. Yet, most observational research on child maltreatment continues to estimate treatment effects without correcting contamination, raising the potential for scientific discovery and replication failures.

The current pre-registered study advances research on the presence and impact of contamination bias in observational child maltreatment research in several important ways. One, we attempted to replicate maltreatment effects on both externalizing behavior problems, such as non-compliance and physical aggression, as well as internalizing behavior problems, such as depressed mood and anxiety, across models that ignored and corrected contamination in a U.S. national probability sample (*N* = 5872) of children involved with child protective services (CPS). Given the already strong evidence for a relation between maltreatment exposure and both externalizing and internalizing behavior problems [16,29], results from the current study demonstrate the impact of ignoring and correcting contamination bias when replicating the direction, statistical significance, and magnitude of maltreatment effects in a nationally representative sample of the child welfare population. Two, we applied a novel, multi-method strategy to detect and correct contamination bias where maltreatment and comparison conditions were established using a substantiation indicator from CPS agencies and where contamination was identified as any caregiver-reported maltreatment in the comparison condition. Caregiver reports are useful for measuring maltreatment especially during infancy and early childhood, when the incidence of maltreatment is highest [30] and the accuracy of self-reports is most limited [31]. Three, we used propensity score analysis [32,33] to approximate the causal effects of maltreatment on child behavior problems three years post-exposure when including a stringent set of covariates. We then conducted supplemental analyses to demonstrate whether the effect of correcting contamination bias is contingent on the quality of covariates selected for propensity score analysis. The primary aim of this study is to evaluate the presence and impact of contamination bias when replicating maltreatment effects on child behavior problems while providing a straightforward approach for correcting such bias to improve replication efforts.

## Materials and methods

Written informed consent and parental permission were obtained from all subjects and/or their parent/legal guardian(s) who participated in the National Survey on Child and Adolescent Well-Being II (NSCAW-II). NSCAW-II study procedures were reviewed and approved by the Research Triangle Institute (RTI) International’s Institutional Review Board prior to data collection. All methods were carried out in accordance with relevant guidelines and regulations. The current study accessed the General Release version of the NCSAW-II data on August 14, 2023, to achieve the current study aims. The research team did not have access to information that could identify individual participants during or after data collection.

### Sample

NSCAW-II is a multi-wave, national probability sample of children and caregivers involved in the U.S. child welfare system [34]. The NSCAW-II sample was generated from 83 counties across 30 states in the U.S. and included sampling weights to improve representation of different types of child maltreatment. Children (*N* = 5872) ranged in age from birth to 17.5 years at Wave 1, which followed the conclusion of an index CPS investigation into alleged child maltreatment. Wave 2 data were collected 18 months after Wave 1 and Wave 3 data were collected 18 months after Wave 2. The final analytic sample for the present study (*n* = 4000; see Table 1) was determined by removing individuals from the larger NSCAW-II sample who: 1) did not have data on the NSCAW-II index case (*n* = 91), 2) were missing data on the NSCAW-II sampling weight or had a sampling weight that was equal to zero (*n* = 1620), or 3) were missing data on caregiver-reported child maltreatment (*n* = 161).

**Table 1:** Sociodemographic information.

	*M (SD)* or *n* (%)		
	No Contamination (n = 60)	Contamination (n = 1503)	Substantiated Child Maltreatment (n = 2437)
**Child Age**	2.88 (4.03)	5.54 (5.06)	4.12 (5.09)
**Child Sex (female)**	27 (45%)	710 (47.24%)	1192 (48.91%)
**Child Race**			
White	36 (62.07%)	837 (57.29%)	1206 (50.91%)
Black	14 (24.14%)	423 (28.95%)	858 (36.22%)
Native Indian/Alaskan Native	6 (10.34%)	149 (10.20%)	210 (8.86%)
Other	2 (3.45%)	52 (3.56%)	95 (4.01%)
**Child Ethnicity**			
Hispanic	22 (36.67%)	412 (27.41%)	780 (32.01%)
**Caregiver Race**			
White	31 (58.49%)	890 (64.26%)	1269 (57.55%)
Black	13 (24.53%)	344 (24.84%)	739 (33.51%)
Native Indian/Alaskan Native	6 (11.32%)	112 (8.09%)	157 (7.12%)
Other	3 (5.66%)	39 (2.82%)	40 (1.81%)
**Caregiver Education**			
No High School Diploma	27 (45.00%)	411 (27.53%)	572 (23.91%)
High School Diploma	21 (35.00%)	674 (45.14%)	941 (39.34%)
Post-High School Training	11 (18.33%)	311 (20.83%)	627 (26.21%)
Bachelor’s Degree or Higher	1 (1.67%)	83 (5.56%)	222 (9.28%)
Other	0 (0.00%)	14 (0.94%)	30 (1.25%)
**Behavior Problems**			
Externalizing	44.12 (11.39)	53.66 (11.67)	52.37 (11.74)
Externalizing – Clinical Level	4 (6.67%)	289 (21.34%)	365 (16.96%)
Internalizing	43.17 (10.26)	52.18 (10.96)	51.05 (11.11)
Internalizing – Clinical Level	2 (3.33%)	228 (16.84%)	303 (14.09%)

**Note.** Percentages represent valid percents of those with available data prior to multiple imputation.

### Measures

#### Child maltreatment.

Child maltreatment and comparison conditions were established based on the results of the index investigation that brought participants into NSCAW-II. If the index investigation substantiated the allegation of child maltreatment, children were assigned to a child maltreatment condition (1 = maltreatment). If the index investigation did not substantiate the allegation, then children were assigned to a comparison condition (0 = comparison). Therefore, the effects of child maltreatment on externalizing and internalizing behaviors evaluated in this study refer to substantiated cases of maltreatment for children involved in child welfare. The substantiation indicator is key, as it reflects the characterization of child maltreatment by independent government agencies masked to the study aim and who followed standardized investigative procedures for officially reporting the incidence and prevalence of child maltreatment in the U.S.

#### Contamination.

Contamination was defined as any caregiver-reported maltreatment at any wave of NSCAW-II for those children in the comparison condition. Caregiver-reported child maltreatment was determined via the Parent-Child Conflict-Tactics Scale [35]. At Waves 1, 2, and 3, caregivers reported on their child’s history of exposure to maltreatment. At each Wave, indicators (0 = no, 1 = yes) were used to determine caregiver-reported maltreatment across the four major types of child maltreatment: psychological, physical, neglect, and sexual. A final indicator represented any caregiver-reported maltreatment at any Wave (0 = no, 1 = yes).

#### Behavior problems.

The six-month frequencies of child externalizing and internalizing behavior problems were measured at Wave 3 via the Child Behavior Checklist [36–38]. Raw scores were originally listed as the outcome variables in the study preregistration, however, to facilitate the interpretation of results, we used *T*-scores at Wave 3 as the outcome variable, which standardize raw scores for child age and sex and have a normative mean of 50 and a standard deviation of 10. Similar patterns of model results were observed when using *T-* and raw scores.

#### Covariates.

Covariates measured at NSCAW-II Wave 1 were selected for inclusion in the primary propensity score analyses if they were hypothesized to not be impacted by child maltreatment and functioned as confounders (i.e., potentially influenced the likelihood of maltreatment exposure and child behavior problems [39]), critical features for covariate selection [40]. These variables were: child age in years, child and caregiver sex, child and caregiver race, and caregiver education level. We then conducted supplemental propensity score analyses to test the quality of covariate selection on estimates of child maltreatment effects before and after correcting contamination. These supplemental analyses retained the covariates listed above but included a wider range of covariates at Wave 1 where the timing of the covariate relative to maltreatment exposure, or whether maltreatment exposure impacted these variables, was unknown and where the covariate functioned as a confounder. This full list of covariates was: child age in years; child and caregiver sex; child and caregiver race; caregiver education level; family income; caregiver-reported domestic violence in the past year; caregiver social support; caregiver drug and alcohol dependence; number of females and males in the household; caregiver service utilization; caregiver child welfare service utilization; child weight status; child’s need for medical, health-related, or mental health services; child Medicaid status; whether the child had been in the household since birth; child receipt of agency-funded services at the time of NSCAW-II enrollment; child delay in physical growth; child service utilization; and child emotional, behavioral, or psychological health concerns.

### Data analysis

Prior to multiple imputation and modeling, an examination of skewness, kurtosis, and potential outliers was undertaken in the analytic sample for child externalizing and internalizing behaviors at NSCAW-II Wave 3. For externalizing behaviors, skewness was 0.24 and kurtosis was 2.78. For internalizing behaviors, skewness was 0.13 and kurtosis was 2.49. Potential outliers were identified as values above or below 3 standard deviations of the sample mean. Eight possible outliers were identified for externalizing behaviors and 4 possible outliers were identified for internalizing behaviors. Model results presented herein include potential outliers that were windsorized to 2 standard deviations of the mean, which did not change the pattern of model results. Missing data on outcomes and covariates (0% − 71.93%) were accommodated via multiple imputation using the *mice* package [41] in R [42] across 25 datasets, each with 100 iterations, using predictive mean matching. The presented results were pooled across 25 imputed datasets following Rubin’s Rules. The sampling weight NANALW23 was used during modeling as data from all three waves of NSCAW-II were included during multiple imputation or model fitting.

Four primary propensity score models tested the study aim of evaluating the presence and impact of contamination bias when replicating maltreatment effects on child behavior problems. Models 1 and 2 estimated the effect for a two-level treatment variable, Substantiated Child Maltreatment (*n* = 2437) relative to a No Substantiated Child Maltreatment comparison condition (*n* = 1563), in models of externalizing and internalizing behaviors, respectively, that ignored contamination. To correct contamination, the No Substantiated Child Maltreatment comparison condition (*n* = 1563) was screened for caregiver-reported maltreatment and partitioned into two subgroups of Contamination (*n* = 1503) and No Contamination (*n* = 60) based on the results of that screen. Models 3 and 4 then estimated the effects of substantiated maltreatment on externalizing and internalizing behaviors, respectively, after correcting contamination using a three-level treatment variable: 1) a Substantiated Maltreatment condition comprised of those children with substantiated child maltreatment (*n* = 2437); 2) a Contamination condition comprised of those children with no substantiated child maltreatment but who had a caregiver report maltreatment (*n* = 1503), and 3) a No Contamination condition comprised of children with neither substantiated nor caregiver-reported maltreatment (*n* = 60). This three-level treatment variable generates three contrasts or group comparisons: Level 1 vs. Level 3, Level 2 vs. Level 3, and Level 1 vs. Level 2. Given the aim and methods of this study, the primary contrast of interest is Level 1, Substantiated Child Maltreatment (*n* = 2437) vs. Level 3, No Contamination (*n* = 60), on externalizing (Model 3) and internalizing (Model 4) behaviors. However, results for all contrasts are reported below. See Table 1 for sociodemographic information on the Substantiated Maltreatment, Contamination, and No Contamination conditions.

For all models, covariates were adjusted using entropy balancing, a form of propensity score weighting that guarantees exact mean balance on the included covariates [43], as implemented in the WeightIt [44] and MatchThem [45] R packages. Entropy balancing was implemented in MatchThem, which interfaces with WeightIt to estimate entropy balancing weights while incorporating sampling weights within each imputed dataset. The average treatment effect (ATE) in the population [32] was targeted, which involves weighting each group to resemble the full sample, which in the current study is a national probability sample of children involved in the U.S. child welfare system. Covariate balance statistics for our primary propensity score models are depicted in Tables 2 and 3. Supplemental propensity score analyses repeated Models 1–4 but with the larger set of covariates to determine whether covariate selection impacted maltreatment effects before and after correcting contamination.

**Table 2:** Covariate balance statistics: Two-level treatment models.

	SMD Unadj.	SMD Adj.	KS Unadj.	KS Adj.
**Covariate**				
Child Age	.057	.000	.070	.049
Child Gender	.067	.000	.067	.000
Caregiver Gender	.015	.000	.015	.000
Caregiver Education	.039	.000	.047	.028
Child Race: Black	.060	.000	.060	.000
Child Race: Other	.004	.000	.004	.000
Caregiver Race: Black	.072	.000	.072	.000
Caregiver Race: Other	.006	.000	.006	.000

Note. SMD = Standardized mean difference. KS = Kolmogorov-Smirnov statistic. Unadj = Unadjusted. Adj = Adjusted.

**Table 3:** Covariate balance statistics: Three-level treatment models.

	SMD Unadj.	SMD Adj.	KS Unadj.	KS Adj.
**Covariate**				
**No Contamination vs. Substantiated CM**				
Child Age	.267	.000	.174	.182
Child Gender	.016	.000	.016	.000
Caregiver Gender	.059	.000	.060	.000
Caregiver Education	.494	.000	.222	.062
Child Race: Black	.146	.000	.146	.000
Child Race: Other	.006	.000	.006	.000
Caregiver Race: Black	.110	.000	.110	.000
Caregiver Race: Other	.031	.000	.031	.000
**No Contamination vs. Contamination**				
Child Age	.339	.000	.168	.164
Child Gender	.085	.000	.085	.000
Caregiver Gender	.045	.000	.045	.000
Caregiver Education	.473	.000	.229	.035
Child Race: Black	.089	.000	.089	.000
Child Race: Other	.009	.000	.009	.000
Caregiver Race: Black	.040	.000	.040	.000
Caregiver Race: Other	.035	.000	.035	.000
**Contamination vs. Substantiated CM**				
Child Age	.070	.000	.074	.052
Child Gender	.069	.000	.069	.000
Caregiver Gender	.014	.000	.014	.000
Caregiver Education	.021	.000	.042	.030
Child Race: Black	.057	.000	.057	.000
Child Race: Other	.005	.000	.005	.000
Caregiver Race: Black	.071	.000	.071	.000
Caregiver Race: Other	.006	.000	.006	.000

Note. CM = Child Maltreatment. SMD = Standardized Mean Difference. KS = Kolmogorov-Smirnov Statistic. Unadj = Unadjusted. Adj = Adjusted.

Because externalizing and internalizing behaviors were represented as *T*-scores, estimates reported below reflect the differences in *T*-scores for those exposed to substantiated maltreatment relative to comparison conditions when contamination is ignored (two-level treatment variable) and when it is corrected (three-level treatment variable). The standardized mean difference (*SMD*) is reported as a measure of effect size when estimating maltreatment effects on externalizing and internalizing behavior problems. *SMD*s were derived by dividing the estimates for maltreatment effects, which represent between-group mean differences in *T*-scores, by the observed whole sample standard deviation for externalizing (*SD* = 11.78) and internalizing behavior problems (*SD* = 11.11), respectively, at NSCAW-II Wave 3.

## Results

### Prevalence of contamination

For those with no substantiated child maltreatment (*n* = 1563), *n* = 1503 had at least one instance of caregiver-reported child maltreatment. Thus, the prevalence of contamination for this study was 96.16%. Table 4 displays the full tabulation of substantiated and caregiver-reported child maltreatment in the analytic sample.

**Table 4:** Substantiated and caregiver-reported child maltreatment.

	No Substantiated Child Maltreatment	Substantiated Child Maltreatment
**No Caregiver-Reported Child Maltreatment**	60	76
**Caregiver Reported Child Maltreatment**	1503	1851

Numbers presented in the table do not sum to the total analytic sample (*n* = 4000) due to the omission of 510 observations. These 510 observations were children with substantiated child maltreatment who were missing data on caregiver-reported maltreatment. Because these children were assigned to the Substantiated Child Maltreatment condition, and not the No Substantiated Child Maltreatment comparison condition that required observed data on caregiver-reported maltreatment to correct contamination, they could be retained in propensity score analyses. However, we omit these 510 observations from the Substantiated Child Maltreatment column presented here because we are not able to tabulate caregiver-reported maltreatment.

### Primary propensity score analyses estimating child maltreatment effects

#### Contamination ignored.

Model 1 showed no statistically significant effect of substantiated child maltreatment on externalizing behavior problems (*b* = 0.28; *p* = 0.746, 95% CI: −1.39, 1.94; *SMD* = .02). Model 2 similarly did not generate a statistically significant effect on internalizing behavior problems (*b* = 0.27; *p* = 0.744, 95% CI: −1.33, 1.87; *SMD* = .02).

#### Contamination corrected.

Model 3 showed a change in the statistical significance and magnitude of maltreatment effects relative to Model 1, where child maltreatment resulted in significantly more frequent externalizing behavior problems with a moderate effect size difference between substantiated child maltreatment and comparison conditions (*b* = 8.72; *p* = .002; 95% CI: 3.17, 14.26; *SMD* = 0.74). Table 5 shows the results of the propensity score analyses for externalizing behavior problems when contamination was and was not corrected. Model 4 also showed a change in the statistical significance and magnitude of maltreatment effects relative to Model 2, where child maltreatment led to significantly more frequent internalizing behavior problems and a large effect size difference between the substantiated maltreatment and comparison conditions (*b* = 9.98; *p* < .001; 95% CI: 6.46, 13.47; *SMD* = 0.90). Table 6 shows the results of propensity score models for internalizing behavior problems when contamination was and was not corrected.

**Table 5:** Propensity score analyses estimating externalizing behavior problems before and after correcting contamination.

	Primary Results	
	Estimate	95% CI
**Contamination Ignored – Model 1**		
Substantiated CM (*n* = 2437) vs. No Substantiated CM (*n* = 1563)	0.28	−1.39 - 1.94
**Contamination Corrected – Model 3**		
Substantiated CM (*n* = 2437) vs. No Contamination (*n* = 60)	8.72**	3.17 - 14.26
Contamination (*n* = 1503) vs. No Contamination (*n* = 60)	8.71**	3.24 - 14.18
Substantiated CM (*n* = 2437) vs. Contamination (*n* = 1503)	0.01	−1.66 - 1.68

**p < .01. CM = Child Maltreatment. Externalizing behavior problems are *T*-scores. Covariates are child age in years, child and caregiver sex, child and caregiver race, and caregiver education. Unadjusted effective sample sizes in 2-group model are No Substantiated CM = 611.55, Substantiated CM = 429.63 while Adjusted effective sample sizes in 2-group model are No Substantiated CM = 613.44, Substantiated CM = 444.84. Unadjusted effective sample sizes in 3-group model are No Contamination = 22.91, Contamination = 589.28, and Substantiated CM = 429.63 while adjusted effective sample sizes are No Contamination = 17.27, Contamination = 589.65, and Substantiated CM = 444.84.

**Table 6:** Propensity score analyses estimating internalizing behavior problems before and after correcting contamination.

	Primary Results	
	Estimate	95% CI
**Contamination Ignored – Model 2**		
Substantiated CM (*n* = 2437) vs. No Substantiated CM (*n* = 1563)	0.27	−1.33 - 1.87
**Contamination Corrected – Model 4**		
Substantiated CM (*n* = 2437) vs. No Contamination (*n* = 60)	9.98***	6.46 - 13.47
Contamination (*n* = 1503) vs. No Contamination (*n* = 60)	9.96***	6.57 - 13.38
Substantiated CM (*n* = 2437) vs. Contamination (*n* = 1503)	−0.01	−1.61 - 1.59

***p < .001. CM = Child Maltreatment. Internalizing behavior problems are *T*-scores. Covariates are child age in years, child and caregiver sex, child and caregiver race, and caregiver education. Unadjusted effective sample sizes in 2-group model are No Substantiated CM = 611.55, Substantiated CM = 429.63 while adjusted effective sample sizes in 2-group model are No Substantiated CM = 613.44, Substantiated CM = 444.84. Unadjusted effective sample sizes in 3-group model are No Contamination = 22.91, Contamination = 589.28, and Substantiated CM = 429.63 while adjusted effective sample sizes are No Contamination = 17.27, Contamination = 589.65, and Substantiated CM = 444.84.

### Supplemental propensity score analyses examining quality of covariate selection

#### Contamination ignored.

Supplemental analyses that repeated Model 1 with the larger set of covariates showed no statistically significant effect on externalizing behavior problems (*b* = −0.25; *p* = 0.799; 95% CI: −2.15, 1.65). Supplemental analyses that repeated Model 2 with the larger set of covariates also did not generate a statistically significant effect for internalizing behavior problems (*b* = −0.31; *p* = 0.745; 95% CI: −2.16, 1.55).

#### Contamination corrected.

Attempts to run supplemental analyses that repeated Models 3 and 4 with the expanded list of covariates did not converge, likely due to the small size of the comparison condition.

## Discussion

A wealth of research has demonstrated a well-established relation between exposure to child maltreatment and an increased risk for more frequent and severe child behavior problems [16,29]. The strength of this evidence has important scientific and practical implications. For example, child behavior problems are among the most common complaints across settings where children access healthcare [46,47] and serve as early, transdiagnostic indicators of future adverse health following maltreatment exposure [48], particularly when levels are clinically elevated [49]. Discovering and replicating maltreatment effects on externalizing and internalizing behavior problems therefore bolsters the scientific foundation of this research that can guide large scale health policy for early screening post-exposure to inform referrals for prevention and treatment.

The current study adds to a growing body of research [23,24,27,28,50] showing a general pattern of results across different samples, populations, and outcomes that contamination is present in child maltreatment research and ignoring it, particularly when the prevalence is high, can result in a failure to detect or replicate the statistical significance and magnitude of child maltreatment effects. The current study also showed that a multi-method strategy for detecting and correcting contamination bias can produce estimates of maltreatment effects that were in the expected direction, statistically significant, and moderate to large in effect magnitude. The ability to replicate results only after correcting contamination bias in this study: 1) is consistent with results from prior research using independent samples with a wide range in contamination prevalence, 2) highlights the contribution measurement plays for detecting contamination in child maltreatment research, and 3) suggests that bias likely exists in estimates generated from studies that do not address contamination. It could be that variation in the prevalence of contamination across studies results in variation in the direction, statistical significance, and magnitude of maltreatment effects, raising the threat of discovery and replication failures for a host of outcomes examined in child maltreatment research, not just child behavior problems.

To our knowledge, this is the first study to identify and correct contamination based on caregiver-reported child maltreatment. Exposure to maltreatment most often occurs in children under the age of two [30], an age range when the accuracy of self-report assessments of maltreatment may be subject to recall and other biases. The opportunity to have an established caregiver prospectively report on maltreatment exposure for infants, children, and adolescents represents a unique opportunity to evaluate the impact of contamination bias on child maltreatment effects for a wider age range. Using caregiver reports resulted in a contamination prevalence of 96 percent in this study. This contamination rate is higher than reported in prior research [23,24,27,28] that relied largely on demographically-matched comparisons sampled from the general population. In contrast, the entire sample in this study was derived from children and families already involved in the U.S. child welfare system. The current sample is therefore best characterized as high-risk for maltreatment where caregivers may be more likely to report maltreatment than what is observed in the general population or what can be substantiated by CPS. This highlights the added sensitivity of a multi-method strategy for detecting contamination while illustrating how the prevalence of contamination may differ across samples at varying risk for maltreatment exposure.

For example, samples drawn from the general population at less risk for maltreatment exposure may have lower prevalences of contamination than what was reported in this study, which may, translate into a smaller degree of contamination bias affecting treatment effect estimates. However, there are two important points to consider here. One, up to 37% of all children in the U.S. are the subject of a CPS investigation into alleged child maltreatment [15]. Because many individuals with a documented history of maltreatment or CPS involvement fail to self-report this information when surveyed [51,52], the risk for contamination still exists even in general population samples and the value of a multi-method strategy for detecting and correcting any resulting bias remains critical. Two, the relation between the degree of contamination in a given study and the resulting impact on maltreatment effect estimates remains unknown. It is entirely possible that as the contamination prevalence increases or decreases, so does its impact on the direction, statistical significance, and magnitude of maltreatment effects. Future research will be needed to evaluate the relation between contamination prevalence and the resulting degree of bias in maltreatment effect estimates across samples at varying degrees of risk for maltreatment exposure.

The present study also evaluated whether the quality of covariates included in propensity score analyses impacted resulting estimates. All primary results were generated from propensity score analyses that included covariates that were confounders and not affected by maltreatment exposure, a critical threshold for covariate selection that lends to greater accuracy and confidence in resulting estimates [40]. Supplemental analyses relaxed this threshold and adopted a wider range of confounders to block potential bias in causal estimates resulting from a failure to include important confounders and that were more likely to be included in other propensity score analyses estimating child maltreatment effects. The inclusion of a wider range of covariates in supplemental analyses did not meaningfully change results in models that ignored contamination, suggesting that the restricted set of covariates was robust in minimizing potential bias resulting from the omission of relevant confounders. Unfortunately, it was not possible to evaluate the impact of including a wider range of covariates in analyses that corrected contamination, likely due to the high prevalence of contamination in this study. The supplemental analyses therefore highlight two key points: 1) the inclusion of additional covariates may not improve estimates of child maltreatment effects when contamination is highly prevalent, and 2) the difficulty of appropriate covariate selection where measurement of variables most often occurs after maltreatment exposure and how the number of covariates selected can have critical implications for models controlling contamination bias, particularly when the prevalence of contamination is high.

There are also important limitations to this study. One, the current study did not examine the impact of contamination bias among different types of child maltreatment and resulting child behavior problems. Therefore, conclusions are limited to broader categorizations of maltreatment exposure. The decision to model a broad category of child maltreatment, instead of individual types, was deliberate and made based on evidence demonstrating that different types of child maltreatment are similarly associated with child behavior problems [53–55]. Ultimately, the primary goal of the current study was to raise awareness about the need to detect and correct contamination bias in research on child maltreatment and using a general category of maltreatment was sufficient for that purpose. Other researchers are encouraged to examine whether the presence and impact of contamination bias varies across child maltreatment types or other dimensions of maltreatment exposure [56]. Two, the current study measured child behavior problems via caregiver-report across all measurement occasions, a range of time where these same outcomes were not assessed by other reporters, such as children. This provided a reliable and valid assessment of both externalizing and internalizing behavior problems [37]. However, caregivers and children may report behavior problems differently [57] and future studies may wish to use a multi-informant approach. Three, we did not examine the concordance of prospective and retrospective reports of maltreatment and how this may affect the impact of contamination in this study. However, our prior research has examined this issue [24], showing concordance of maltreatment exposure across confirmed records and self-report surveys significantly predicted child behavior problems across child development after contamination was corrected. This finding was in contrast to results showing that discordance, specifically a condition where there were confirmed records of maltreatment but no self-reports and another condition where there were self-reports of maltreatment but no confirmed records, did not significantly predict child behavior problems after correcting contamination. Four, there is no one gold-standard measure of assessing child maltreatment, including the official case reports and caregiver-reports used in this study but also including self-reports. This means that contamination is likely to exist in any sample or research design when only one measure of child maltreatment is used in a study (i.e., contamination cannot be detected) and that the use of multiple methods may still not capture all contamination. While the multi-method strategy in the current study detected contamination at a prevalence of 96%, other measures such as administrative timing data or multi-informant assessments across settings may provide additional flexibility for detecting contamination as part of a multi-method and time-varying assessment strategy. Five, the high prevalence of contamination, combined with the use of sampling weights to ensure generalizability to the broader child welfare population, resulted in a small raw sample size as well as small adjusted and unadjusted effective sample sizes for propensity score analyses with the no contamination condition. Thus, it is important to note that the no contamination comparison condition was impacted by significant weight variability. Replication of this work that evaluates the relation between contamination prevalence and resulting degree of bias in maltreatment effect estimates is needed. Finally, this study did not account for genetic factors that may play a role in exposure to maltreatment or the onset and course of child behavior problems [58,59]. Future research in this area could estimate such genetic liability, either in terms of gene-environment correlations or heritability estimates, and include it within a list of covariates used to balance maltreatment and comparison conditions in propensity score models.

## Conclusions

The results of this study suggest the estimation of child maltreatment effects is biased by the presence of contamination, a phenomenon that can increase replication failures for child behavior problems and likely other outcomes [27,28]. The present study makes significant advancements in the estimation of maltreatment effects by reporting results from a multi-method strategy for identifying and correcting contamination that replicated maltreatment effects on child behavior problems reported in prior research. Notably, ignoring contamination did not replicate prior findings. Not only is contamination prevalent in child maltreatment research but correcting it can address resulting bias and improve discovery and replication of findings across outcomes. The methods for correcting contamination reported here may also aid the estimation of treatment effects in observational research outside the area of child maltreatment, as contamination can occur in any study comparing treatment and control groups [60].
